# Texture-Based Neural Network Model for Biometric Dental Applications

**DOI:** 10.3390/jpm12121954

**Published:** 2022-11-25

**Authors:** Omnia Saleh, Kosuke Nozaki, Mayuko Matsumura, Wataru Yanaka, Hiroyuki Miura, Kenji Fueki

**Affiliations:** 1 Department of Masticatory Function and Health Science, Graduate School of Medical and Dental Sciences, Tokyo Medical and Dental University, Bunkyo-ku, Tokyo 113-8510, Japan; 2 Department of Advanced Prosthodontics, Graduate School of Medical and Dental Sciences, Tokyo Medical and Dental University, Bunkyo-ku, Tokyo 113-8510, Japan

**Keywords:** personalized dentistry, artificial intelligence, texture, deep learning, convolutional neural network

## Abstract

Background: The aim is to classify dentition using a novel texture-based automated convolutional neural network (CNN) for forensic and prosthetic applications. Methods: Natural human teeth (*n* = 600) were classified, cleaned, and inspected for exclusion criteria. The teeth were scanned with an intraoral scanner and identified using a texture-based CNN in three steps. First, through preprocessing, teeth images were segmented by extracting the front-facing region of the teeth. Then, texture features were extracted from the segmented teeth images using the discrete wavelet transform (DWT) method. Finally, deep learning-based enhanced CNN models were used to identify these images. Several experiments were conducted using five different CNN models with various batch sizes and epochs, with and without augmented data. Results: Based on experiments with five different CNN models, the highest accuracy achieved was 0.8 and the precision was 0.8 with a loss value of 0.9, a batch size of 32, and 250 epochs. A comparison of deep learning models with different parameters showed varied accuracy between the different classes of teeth. Conclusion: The accuracy of the point-based CNN method was promising. This texture-identification method will pave the way for many forensic and prosthodontic applications and will potentially help improve the precision of dental biometrics.

## 1. Introduction

Biometric identification has immense importance in forensics as well as personalized medicine [1,2]. In identification, several parts of the human body are used [3]. Human enamel is the hardest tissue on earth and is extremely resistant to elevated temperature and chemical changes [4,5,6], making dental identification an essential alternative to soft-tissue methods [7,8].

Several features of the teeth may be used for identification such as contours, dimensions, arch size, bite marks, (estimated) dental age, dental restorations, and teeth development. These can be used alone or in combination [4,8,9,10]. Ameloglyphics, the study of teeth patterns, has been proposed as a form of biometric identification feature like fingerprinting and iris detection [3,5]. Natural teeth exhibit individual textural features, and the exact patterns of these features are unique [11,12]. Precisely recording these details makes it feasible to use these patterns for biometric applications [3]. Methods of recording dental prints include the peeling technique, recording with silicon impressions or celluloid acetate films, and automated biometric analysis [3,5].

Digital transformations in dentistry are becoming the new standard in clinical practice. One application of digital dentistry is creating digital impressions using oral scanners, which have proven to be cost-effective, time effective, and highly accurate [13]. It provides a comfortable option for the patient, without harmful side effects, regardless of repeated use [14,15]. According to recent studies, intraoral scanners are accurate to within a few microns [13,16].

The concept of personalized treatment and biomimetically designed dental prostheses is gaining popularity in dentistry, and artificial intelligence (AI) currently plays a significant role [15,17,18]. In prosthodontics, although digital production can decrease the time and cost of dental treatment, it is challenging to reproduce the unique morphological features of teeth because of limitations in computer design and construction methods [18,19]. This has led to the introduction of the concept of Digital Dental Passport, which is the application of an individual’s dental library that is easily retrieved when needed [18].

Convolutional neural networks (CNN) have been implemented in many image processing applications [20,21,22]. Deep learning is accurate in the identification and classification of radiographs, as shown in previous research [23,24,25]. However, to date, limited dental studies have been conducted on the classification of 3D (3 Dimensional) scanned images [14,26,27].

Texture analysis plays a key role in computer vision, especially object detection. It has advantages if used individually or in combination with other methods such as facial anatomy of the subject, which is increasingly comparable with digital software after digital acquisitions of intraoral impressions and facial scanners [28,29]. The Discrete Wavelet Transform (DWT) is a method used for feature texture extraction, using translations and discrete wavelet scales [30]. The DWT method is used for an efficient and quick de-noising of the signal and its implementation is also considered computationally efficient [31]. This study focuses on the development of a novel texture-based biometric application for scanned dentition, a process based on the DWT extraction feature for classification.

## 2. Materials and Methods

### 2.1. Teeth Collection, Scanning, and Classification

Extracted natural teeth of unknown origin (*n* = 600) were sourced from the Maxillo-facial Anatomy Department of the Tokyo Medical and Dental University. Sample size per group was calculated based on previous studies; the expected *p* was 0.02 and the desired precision (*d*) was 0.05. A total of 31 samples per class are required when the population is infinite using the following Equation (1) [32].

(1) $$n^{\prime }=\frac{NZ^{2}P\left(1-P\right)}{d^{2}\left(N-1\right)+Z^{2}P\left(1-P\right)}$$

*n*′ = Sample size with finite population correction

*N* = Population size

*Z* = Z statistic for a level of confidence

*P* = Expected proportion (in proportion of one)

*d* = Precision (in proportion of one).

The teeth were cleaned using an ultrasonic scaler (Varios 970, NSK, Tokyo, Japan) at 28–32 KHz frequency to remove any debris. Subsequently, they were scanned with an intraoral scanner (Trios 3, 3Shape, Copenhagen, Denmark). Furthermore, the teeth were aligned to view their frontal surface. Then, using design software (Autodesk Meshmixer, Mill Valley, CA, USA), images of the frontal surfaces of the teeth were captured in both PNG and JPG formats. The images were classified into nine groups [12,33] and labeled from 0 to 8, as shown in Table 1.

**Figure 1:**
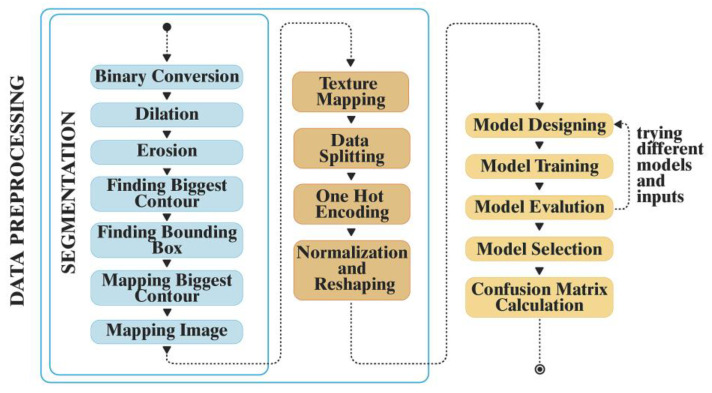
Process of the convolutional neural network (CNN)-based AI model used in the study. First is data preprocessing, which includes segmentation, followed by texture mapping, and finally model designing and evaluation.

Python was used in this study [34] and the proposed method consisted of several steps as presented in Figure 1.

### 2.2. Preprocessing

After classification, the tooth images were preprocessed by converting them into binary images. Morphological operations, including erosion and dilation, were used to remove outliers. Erosion and dilation operations were based on kernel size and used as structuring elements to reduce the size of the input image. Similarly, dilation increased the size of the input image based on kernel size. A kernel size of 10 × 10 was used in the proposed method. The erosion and dilation of the binary image was calculated based on Equations (2) and (3), respectively, where *A* represents the original binary image and *B* represents the kernel. The front-facing tooth image was selected after finding contours in the binary image.

(2) $$A⊖B=z\in E|B_{z}$$

(3) $$A⊕B=\underset{b\in E}{\cup }A_{b}$$

### 2.3. Extracting Textural Features Using DWT

In this study, wavelets were derived because they contain certain features useful in image processing. Wavelet coefficients were used as feature vectors for image classification. Using DWT, a one-variable function was converted into a two-variable function: scale and translation. Wavelet coefficients were calculated as discrete values based on the power of two, as shown in Equation (4).

(4) $$W\left(j,k\right)=\sum j\sum k*x\left(k\right)2^{-\frac{j}{2}}\Psi 2^{-j}\left(n-k\right)$$

In the above equation, the discrete function *x* calculated the weighted sum of wavelets and was added to the coarse component. Furthermore, the coarse approximation was decomposed by a low pass followed by high pass iterations. The calculations of the approximation and detailed components are shown in Equations (5) and (6).

(5) $$a_{j+1}\left[k\right]=\overset{+\infty }{\underset{m=-\infty }{\sum }}l\left[m-2k\right]a_{j}\left[m\right]$$

(6) $$d_{j+1}\left[k\right]=\overset{+\infty }{\underset{m=-\infty }{\sum }}h\left[m-2k\right]a_{j}\left[m\right]$$

**Figure 2:**
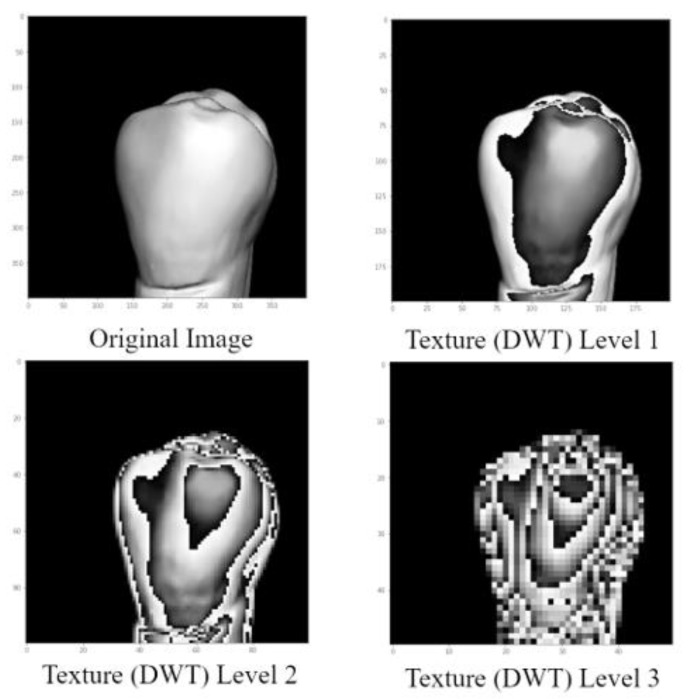
Levels of discrete wavelet transform (DWT) texture extraction. Experiments of three different texture levels (level 1, level 2, and level 3), to reach the highest accuracy.

In DWT, experiments were done using three distinct levels: level 1, level 2, and level 3. Due to its superior accuracy, level 2 was selected for the CNN model. The outcome of the DWT texture-based image for level 2 is shown in Figure 2.

### 2.4. Deep Convolutional Neural Networks for Classification

Different deep learning models with varying numbers of convolutional, pooling, and dropout layers were tested to find the best possible model. Data augmentation was performed to increase the data size and variation. Augmentation was performed with randomly selected values for rotation, zoom level, width/height shift, and shear.

**Figure 3:**
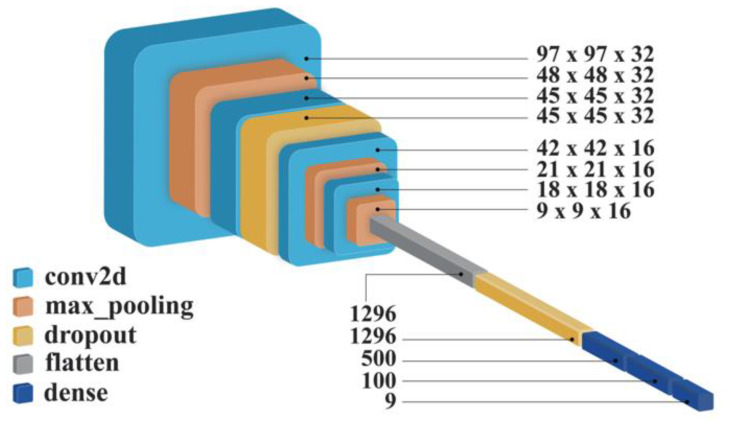
Architecture of the proposed novel convolutional neural network (CNN) model, which includes four convolutional layers, three pooling layers, two dropout layers, and flatten and dense layer.

In CNN, hyperparameter tuning is an optimization problem. By using a cross-validation set along with the trial-and-error method, it was possible to tune the numbers of convolutional layers, pooling layers, dropout layers, and dense layers. It was found that an optimized CNN model comprising 14 layers with 728,789 parameters yielded the optimal results. The architecture of the proposed CNN model is presented in Figure 3.

In the experiments, the tooth image data were split into training and validation sets at an 80:20 ratio. To enhance the size of the training set and provide better validation, the training data were further augmented. The performance of the proposed model was evaluated using accuracy and the confusion matrix. Accuracy denotes the percentage of correct selection. For example, if the accuracy of the model is 50%, it implies that our model is capable of correctly identifying the class of 50% of the teeth samples. Furthermore, to reach a detailed picture, a confusion matrix was constructed to represent different performance-related measures against each class. Using the class-wise accuracy obtained from the confusion matrix, precision or recall was determined.

## 3. Results

### 3.1. Experimental Results and Improvement Steps

**Figure 4:**
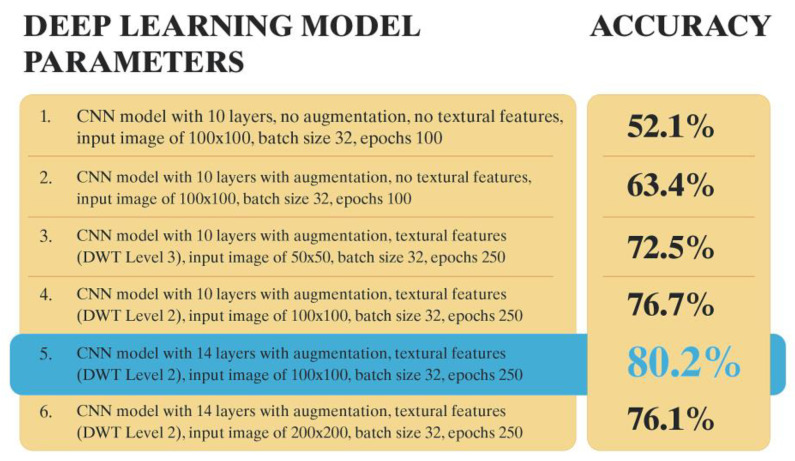
Comparison of deep learning configurations with different parameter combinations, with and without augmentation. Configuration 5 achieved the highest accuracy.

To achieve a good combination of hyperparameters and obtain the best performance from the model, several experiments were conducted based on their relevant performance (in terms of accuracy). We selected the top six for discussion in this section. Different configurations of the proposed model were built based on batch size, number of epochs, and the binary condition of being with or without augmented data. All six configurations and their accuracies are presented in Figure 4. The highest accuracy of 0.8 (80%) was achieved with configuration 5, with a loss value of 0.9 for a batch size of 32 and 250 epochs. There are a few key learning points discussed in Appendix A. Conclusively, the best accuracy was obtained using 14 layers with data augmentation, DWT level 2 textural features, and an appropriate image size as shown in Figure 4. Detailed results are featured in Appendix A Table A1.

### 3.2. Confusion Matrix

**Figure 5:**
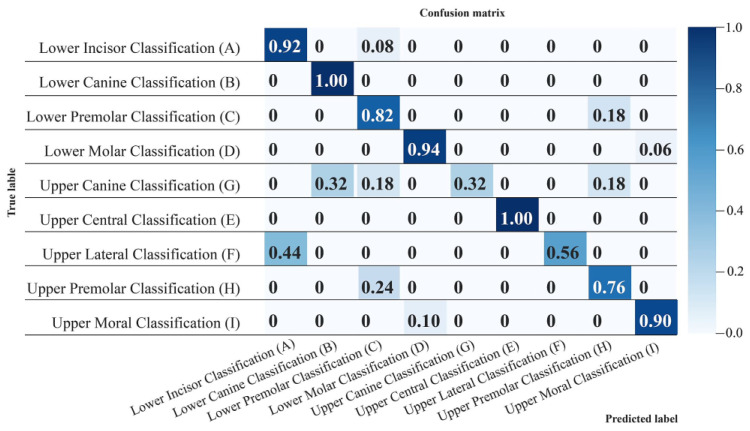
Confusion Matrix of the convolutional neural network (CNN)-based AI model for all teeth classes (Class A to H). The correct classification is presented in the diagonal grids, with classes B and E showing the highest accuracy of 1 (100%) and Class G showing the lowest accuracy of 0.32 (32%).

For clarity and to determine class-wise performance, a confusion matrix of the best performing model is shown in Figure 5. The numbers zero to eight represent tooth classes A to H, respectively.

As shown in Figure 5, the upper central and lower canine tooth classes were detected and classified with the highest degree of accuracy among all classes (100%); whereas the upper canine (32%) and upper lateral (56%) displayed the lowest accuracy.

## 4. Discussion

This study aimed to demonstrate the feasibility of using an automated texture-based model classier for dentition. A subjective identification and classification of dentition could lead to errors, is time-consuming, is limited by the lack of experienced manpower [11,24], and previous studies on dental classification have mainly referred to a single class of teeth [11]. Currently, machine learning texture-based automated systems and software tools can perform fingerprint recognition, facial recognition, and iris scanning that enable reliable biometric applications [35]. Thus, the incorporation of textural features-based deep learning methods in teeth classification of all types presents a solid alternative to subjective identification methods.

In this research, a complete set of extracted natural teeth was considered for two main reasons. First, because individual changes may occur in natural teeth, such as restorations and loss [18], and in forensics, it is feasible to identify whether a particular class of teeth is more amenable than another. Therefore, it is recommended to refer to several teeth classes. Second, it is useful to compare the textural feature uniqueness of several teeth, especially for prosthetic applications.

The intraoral scanner used in this research could accurately capture tooth details of less than 10 microns [13]. The digital storage of dental data was facilitated by the introduction of scanners [14,27]. Studies using scanned dental arches for biometric applications are still limited but rapidly increasing [2]. Recent studies suggest the use of occlusal surfaces of posterior teeth for classification and identification, reporting promising results [11,14]. However, it was also suggested that other teeth be included for future research [14].

In prosthetic treatment, teeth morphology generated from digital libraries cannot replicate an individual patient’s morphology. The duplication of the original tooth or mirroring of the contralateral tooth, if present, could be a solution, but it will require the correct 3D tooth position [19]. Therefore, the creation of personalized digital dental libraries and the associated use of AI identification could help implement the customization concept in digital prosthodontic design [17,18].

In this study, a fully automated method was proposed and achieved, but with some outliers. Data preprocessing is vital for any machine leaning process. In some conditions, it can correct defects that might otherwise affect the learning process, such as noise, omissions, and the presence of outliers [36]. Frequently, preprocessing makes the data less complex and enhances the training of the learning model. Convergent to the traditional segmentations of models, the capacity for abstraction in CNNs enables them to operate in a legitimate, high-element space that minimizes the demand for manually capturing data. However, it is still crucial to have suitable preprocessing to enhance the quality of the learning process [37].

DWT is a well-known mathematical method used for extracting textural features from images [31,38]. Developed in the 1980s for decomposing a signal with finite energy in the spatial domain to a set of orthogonal functions defined in the modular spatial domain [39]. It decomposes signals in the time-frequency domain into basic functions called wavelets.

The algorithms proposed for deep learning architectures have been successful in various fields, such as image restoration and speech and image recognition [40]. It is evident through this research that deep learning architectures in CNNs have been effective. An increased number of hidden layers led to a higher rate of recognition, data augmentation, and the utilization of textural features. Moreover, a smaller batch size was used to reduce memory usage [41]. Although it produced better results in this research, the benefits of a smaller batch size depended upon the number of output classes. Therefore, it is recommended to use a batch-size at least twice the size of the input classes.

The results showed that having more hidden layers enhanced the recognition rate but increased computational time since training time is directly proportional to architecture size [42]. The number of epochs was adjusted with the help of the cross-validation set, and the training process was stopped when the loss started increasing on the validation set. This was done to avoid overfitting, which occurs when the training error is exceptionally low, but the validation error is high. The tooth classification experiments were performed with 100, 150, 250, and 300 iterations; the optimal performance was observed at 250, based on the foregoing criterion.

When accuracy needs to be visualized for unbalanced datasets, a confusion matrix is used to evaluate performance [43]. According to the results, the overall accuracy of the model was 80%. Liu et al.; proposed a Haar Wavelet Transform for the classification of only four classes of teeth utilizing CBCT root sections, and they achieved similar results [44]. The accuracy for identification of the upper central incisor was 100% since it is the most difficult to replicate, and the variation in its microanatomy and surface texture increases its uniqueness. In addition, the accuracy of the lower canine was also 100%, which is superior to previous classification studies with CBCT images [44]. Conversely, the upper canine had the lowest accuracy of 32%, which is justified because this class had the fewest number of samples with a size of only 34 teeth. The upper lateral also has less surface texture than other anterior teeth [12,45], which could be a reason for its lower accuracy as compared with the other classes, where the upper lateral class was confused with the lower anterior class. In a previous study on texture ocular recognition, superior performance was achieved with 50 sample photos [35]; however, this data size may not be comparable to dentition.

The proposed CNN method showed promising overall performance for the incorporation of data augmentation and texture extraction features. Using DWT significantly improved CNN performance. Furthermore, the intraoral scanner served as a convenient tool for recording teeth details with high accuracy. A limitation of this study is that progressive recording has not been tested since these precise records might require periodic updates to overcome any surface loss [5,18]. In the future, this method will be investigated with full arch scans and an automated system will be developed for sorting dental charts. In conclusion, texture-based classification can greatly improve biometric, forensic, and personalized dental applications.

## 5. Conclusions

Texture-based automatic classification is a promising biometric application. The effectiveness of the novel CNN classification model based on the Discrete Wavelet Transform was validated with an accuracy of 80%. This proposed method has potential in forensics and prosthodontics. Future research will be adopted for in-vivo full arch studies.

## Figures and Tables

**Table 1:** Tooth classification and number of samples.

Label	Tooth Class Name		Number of Images
0	Lower Anterior	(A)	64
1	Lower Canine	(B)	87
2	LowerPremolar	(C)	77
3	Lower Molar	(D)	71
4	Upper Centra	(E)	75
5	Upper Lateral	(F)	49
6	Upper Canine	(G)	34
7	UpperPremolar	(H)	80
8	Upper Molar	(I)	63

## Data Availability

Data are available on request but may be restricted for reasons such as privacy or ethical concerns.

## Appendix A

There are a few key learning points that are discussed below:

1. The impact of data augmentation can clearly be seen from the comparison of configuration 1 and 2. The rest of the parameters are the same; however, the only difference is data augmentation that results in improvement of around 11.3% in accuracy.
2. The role of textural features is evident from the comparison with configuration 2 and 4. The accuracy of configuration 4 is 13.3% higher than the accuracy of configuration 2.
3. The DWT level is also playing a crucial role. For example, configuration 3 uses DWT level 3 and configuration 4 uses DWT level 2. As can be seen from their comparative results, the accuracy of configuration 4 is 4.2% higher than the accuracy of configuration 3. One more thing to be noted is the image size is larger in configuration 4 and still configuration 4 is performing better. We can conclude that DWT level 2 is a better performer in this case.
4. Another important performance element is the number of layers. The only difference between configurations 4 and 5 is the number of layers they are using. As can be seen from the table, the accuracy of configuration 5 is 3.6% higher than the accuracy of configuration 4 and that is due to 4 extra layers. Hence, it concludes that layers are also improving accuracy.
5. One last observation that we can extract from this data is the poor impact of image size. If we compare accuracies of configuration 5 and 6 then we find that a higher image adversely affects the performance of the classifier.

The table shows the Precision, Recall. F-Measure and Accuracy score. The average value for the precision was 0.8, for recall it was 0.8, the average value for f -measure was 0.8 and the overall accuracy average value was 0.8.

**Table A1:** Classifier evaluation metrics: Precision, Recall, F-measure and overall accuracy for each Featured class.

Evaluation Metrics/Teeth Classes	Precision	Recall	F-Measure	Accuracy
Lower Incisor Classification (A)	0.9	0.8	0.9	0.9
Lower Canine Classification (B)	0.9	0.8	1	1
Lower Premolar Classification (C)	0.8	0.8	0.8	0.8
Lower Molar Classification (D)	1	0.8	0.9	0.9
Upper Canine Classification (G)	0.3	0.3	0.4	0.3
Upper Central Classification (E)	0.9	1	1	1
Upper Lateral Classification (F)	0.5	1	0.6	0.6
Upper Premolar Classification (H)	0.7	0.7	0.8	0.8
Upper Molar Classification (I)	0.9	0.8	0.9	0.9
Average	0.8	0.8	0.8	0.8
